# Neural computation of visual imaging based on Kronecker product in the primary visual cortex

**DOI:** 10.1186/1471-2202-11-43

**Published:** 2010-03-26

**Authors:** Zhao Songnian, Zou Qi, Jin Zhen, Yao Guozheng, Yao Li

**Affiliations:** 1 LAPC, Institute of Atmospheric Physics, Chinese Academy of Sciences, Beijing 100029, China; 2 Department of Computer Science, Beijing Jiaotong University, Beijing 100044, China; 3 fMRI Center of Brain's function, Beijing 306 Hospital, Chinese People's Liberation Army, Beijing 100101, China; 4 College of Information Science, Peking University, Beijing 100871, China; 5 State Key Laboratory of Cognitive Neuroscience and Learning, School of Information Science and Technology, Beijing Normal University, Beijing 100875, China

## Abstract

**Background:**

What kind of neural computation is actually performed by the primary visual cortex and how is this represented mathematically at the system level? It is an important problem in the visual information processing, but has not been well answered. In this paper, according to our understanding of retinal organization and parallel multi-channel topographical mapping between retina and primary visual cortex V1, we divide an image into orthogonal and orderly array of image primitives (or patches), in which each patch will evoke activities of simple cells in V1. From viewpoint of information processing, this activated process, essentially, involves optimal detection and optimal matching of receptive fields of simple cells with features contained in image patches. For the reconstruction of the visual image in the visual cortex V1 based on the principle of minimum mean squares error, it is natural to use the inner product expression in neural computation, which then is transformed into matrix form.

**Results:**

The inner product is carried out by using Kronecker product between patches and function architecture (or functional column) in localized and oriented neural computing. Compared with Fourier Transform, the mathematical description of Kronecker product is simple and intuitive, so is the algorithm more suitable for neural computation of visual cortex V1. Results of computer simulation based on two-dimensional Gabor pyramid wavelets show that the theoretical analysis and the proposed model are reasonable.

**Conclusions:**

Our results are:

1. The neural computation of the retinal image in cortex V1 can be expressed to Kronecker product operation and its matrix form, this algorithm is implemented by the inner operation between retinal image primitives and primary visual cortex's column. It has simple, efficient and robust features, which is, therefore, such a neural algorithm, which can be completed by biological vision.

2. It is more suitable that the function of cortical column in cortex V1 is considered as the basic unit of visual image processing (such unit can implement basic multiplication of visual primitives, such as contour, line, and edge), rather than a set of tiled array filter. Fourier Transformation is replaced with Kronecker product, which greatly reduces the computational complexity. The neurobiological basis of this idea is that a visual image can be represented as a linear combination of orderly orthogonal primitive image containing some local feature. In the visual pathway, the image patches are topographically mapped onto cortex V1 through parallel multi-channels and then are processed independently by functional columns. Clearly, the above new perspective has some reference significance to exploring the neural mechanisms on the human visual information processing.

## Background

Human vision can be considered as a perfect image information processing device, it can easily recognize object's position, size, and orientation, pose in space, and so on. For a long time, visual scientists, computational neuroscientists, image processing experts and computer vision researchers make great effort to explore the neural mechanism of humans' remarkable visual abilities or how the retina image is represented in the primary visual cortex, which is related with the following two questions: what kind of neural computation is actually performed by the primary visual cortex, and how this is described mathematically.

It is well known that there is a one-to-one topographical mapping between retina and cortex V1, which determines projecting relations in visual space and represents some transformations from retina to cortex V1 [1-16]. Currently, it is believed that responses of neurons in cortex V1 can be simulated by a set of tiled spatio-temporal filters array. So the function of cortex V1 is to make a spatial local Fourier Transform. Theoretically, these filters involve many processes about spatial frequencies, orientations, motion and velocities (frequencies in temporal space) [17-23].

Is this notion consistent with the actual biological visual information processes? Research in neurobiology indicates that the metabolism and decay of neurons do not affect the visual function. Every neuron performs a simple ON-OFF function and transfers the information through spikes. So a dead neuron can easily be replaced by other nearby neurons [3,24,25]. In case of a complicated function, this replacement would be difficult. Therefore, the simplicity of algorithms not only reduces error rates to the minimum, but also guarantees repeatability and stability, i.e. robustness.

Complex computations can be carried out by parallel computations of neuronal groups with high efficiency. So we believe that the actual computations in retinal image must be simple, repeatable and robust and be performed by individual neurons at the system level and by neuronal groups with high efficiency. They are obvious requirements for neural computations in V1.

Then, how is the topographical mapping from retina to V1 be realized? Many neurobiological experiments and visual computational models show that when every primitive (edge, corner and contour) in the visual image finds matches in the receptive fields densely distributed on V1, only the neurons whose frequencies and orientations are similar to those of the primitive fire [26]. Therefore, the patterns of the fired neurons correspond to the primitives in the visual image, which may be represented by a topographical mapping, reflecting the adjustment of the visual image to neurons in V1, and reflecting distributive and parallel visual information processing between retina and V1[27,28]. In this paper, we discuss the mathematical representation of this information processing and use the normalized matching measure (i.e. energy function) to measure the matching extent [29].

This paper proposes a model of neural information processing based on the topographical map in place of Fourier Transform. In this model, the functional columns are not considered as sets of tiled filters, but basis elements of the visual information, including orientation selectivity and feature matching [2,30-32]. The visual image carried by spike trains is processed by Kronecker product with functional columns in V1. So synchronously parallel computations on the whole image can be performed by receptive field-to-receptive field rather than by pixel-to-pixel, and be represented by Kronecker product between matrixes. Its complexity is greatly reduced as compared with Fourier transform and other matrix computations [33,34]. What is more, this algorithm can simulate stimulations of the elements in the visual image to cortical neurons as the embodiment of simple neural functions. The aggregative computation based on simple functions is one of plausible approaches of the visual cortex in realizing topographical mapping.

Numerical simulations are carried out to justify the above notions. In our experiments, receptive fields of neurons in V1 are simulated by hierarchical Gabor functions [35-40]. Visual image is the feature image of Lenna processed by pre-processing (filtered) in front of the pathway. Results of our algorithm are consistent with theoretical expectations.

## Results

The visual pathway ('what' pathway [41]) from retina across LGN to V1 is modelled. The following discussions are focussed on: 1. the optimal detection in V1 of retinal image *R*(*x*, *y*); 2. the optimal matching between *R*(*x*, *y*) and firing pattern of neuronal groups in V1; 3. Kronecker product obtained by optimal detection and matching; 4. determination of kernel function *G*(*x*, *y*); 5. realization of Kronecker product; 6. numerical experiments and discussions.

### Image division and cortical response

**Figure 1:**
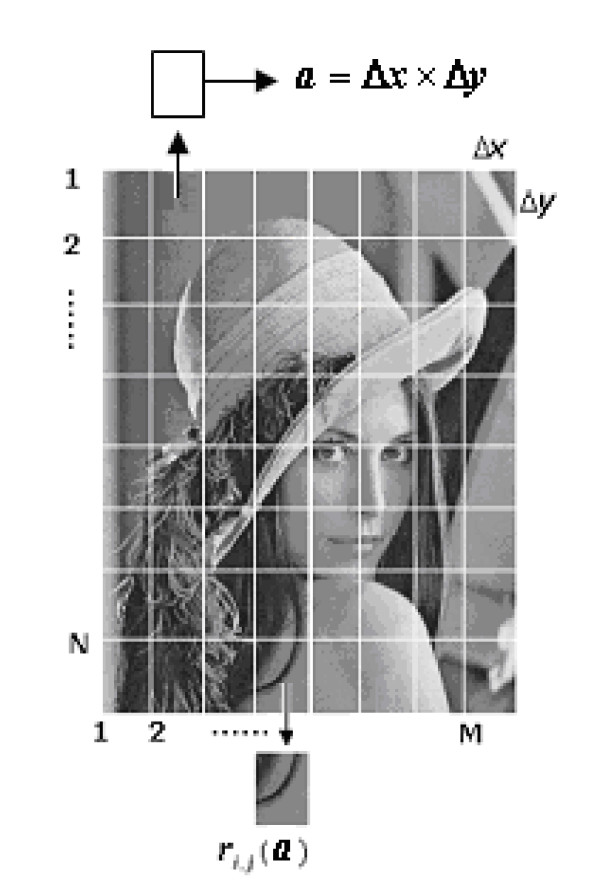
**Visual image *R *(*x, y*) is divided into *M *× *N *local patches according to a ganglion cell's receptive field**.

As a first step of visual perception, external stimuli form a retinal image *R*(*x*, *y*). In the retina, a ganglion cell receives inputs from about 10^3^~10^4 ^photo-receptor cells. If the size of a ganglion cell's receptive field is *a *= Δ*x *× Δ*y*, the total imaging area of *R*(*x*, *y*) in the retina is *A*, which is divided into *M *× *N *patches with the same size *a *as that of a ganglion cells receptive field, namely *A *= (*M *× *N*)*a*. Here *a *is referred to as sub-region, therefore, *a *indicates only its size; and the partially small image covered by the area *a *is called image unit or image primitive, and expressed by *r*(*a*). In this way, a patch or the image primitive at the *i *line and the *j *column can be denoted as *r*_*i*, *j *_(*a*)(*i *= 1,2,⋯, *M*; *j *= 1,2,⋯, *N*), and the entire image included in the area *A *is also expressed by *R*(*A*), as shown in Figure 1. That is to say, the whole image *R*(*A*) can be also formed from (*M *× *N*) patches by means of orderly putting patches together, i.e. .

Figure 1. Visual image *R*(*x*, *y*) is divided into *M *× *N *local patches according to a ganglion cell's receptive field.

This division involves two aspects. First, every patch *r*_*i*,*j*_(*a*) contains local features (such as shapes of receptive fields and orientations) at (*i*, *j*) with area (Δ*x *× Δ*y*), while a pixel at (*i*, *j*) only contains intensity information. Second, in the parallel multi-channel vision system, every channel only deals with a local patch. Obviously this division is consistent with parallel and multi-channel properties of a vision system, and in a topographical mapping any patch can be located in the retina, so the neural processing based on functional columns can be realized and the corresponding mathematical description is possible. A patch *r*_*i*,*j*_(*a*) will activate the corresponding ganglion cell and output a coded firing spike train. This is transferred across LGN into V1 as a topographical mapping. Then, the firing spike train is decoded and the image represented by *r*_*i*,*j*_(*a*) is restored. A Kronecker product of the restored image with functional columns *B*_*k*,*l*_(*s*) in V1 [40,41,33] leads to firing of neurons in receptive fields having similar orientation and bandwidth (that is so-called firing under a preferential stimulus). We denote the firing pattern of one singular neuron (or simple cell) as *ϕ*_*i*,*j*_(*b*), where *b *denotes the area of the receptive field of the neuron. Since, *A*, the retinal image area, will be enlarged in the cortex V1 [42], for simplicity, with a magnification factor *z *= 2^*h *^(*h *= 0,1,2,⋯); if the area of image on the cortex V1 is denoted by *B*, we have *B *= *zA *= 2^*h *^*A*, *b *= *za *= 2^*h *^*a*. In this way, the spatial sum of all signals *ϕ*_*i*,*j*_(*b*) in an orderly manner will form the overall firing pattern, that is , which represents a reconstruction of the retinal image *R*(*A*) on V1.

### Optimum detection in V1

For a single neuron in the functional columns, its receptive field consists of orientation, band-pass and spatial location [43,44], with a strong selectivity with respect to visual image *R*(*x*, *y*) topologically mapped from the retina. When some patch *r*_*i*,*j*_(*a*) in visual image *R*(*x*, *y*) shows properties at specific orientation and specific frequency similar to some receptive field *g*_*i*,*j*_(*b*), the corresponding neuron will respond strongly. In other words, when a local patch coincides with the receptive field of a neuron at its sensitive orientation and sensitive frequency, the neuron fires most strongly, which means a detection and matching of functional columns in V1 to local retinal patch's features *r*_*i*,*j*_(*a*), as a random process, in other words, which is a detection and matching in a random process. Therefore, this process can be expressed as [45](1)

where *r*_*i*,*j*_(2^*h *^*a*) is a patch from input image *R*(*A*) containing basic features such as orientation, edge, and contour within the receptive field. Here *n*_*i*,*j *_is Gaussian white noise with zero mean and variance . It is assumed here that the noise environment in different visual pathways is the same, i.e. *n*_*i*,*j *_is the same. *α*_*i*,*j *_is weight coefficient that can reflect excitation strength of an image primitive to neuron *g*_*i*,*j*_(*b*). We want to know what value of *α*_*i*,*j *_can strongly activate neuron *g*_*i*,*j*_(*b*) to fire. Obviously, *α*_*i*,*j *_is an unknown estimated coefficient. If we make *K *observations on the random process (1), in other words, we carry out *K *sampling on the random process (1), then formula (1) can be written in the following general form(2)

According to maximum likelihood estimation and least mean square error rule [45,46](3)

The optimum estimation of *a*_*i*,*j *_is(4)

where  is a ratio coefficient.

We can see *a*_*i*,*j *_reaches its optimum value  when image patch *r*_*i*,*j*_(*a*) coincides with receptive fields feature *g*_*i*,*j*_(*b*), i.e. *a*_*i*,*j*_*r*_*i*,*j *_(2^*h *^*a*) = *g*_*i*,*j*_(*b*). So,  can be taken as the measure for matching extent between neuronal receptive field *g*_*i*, *j*_(*b*) and patch *r*_*i*,*j*_(*a*) in image *R*(*A*).

**Figure 2:**
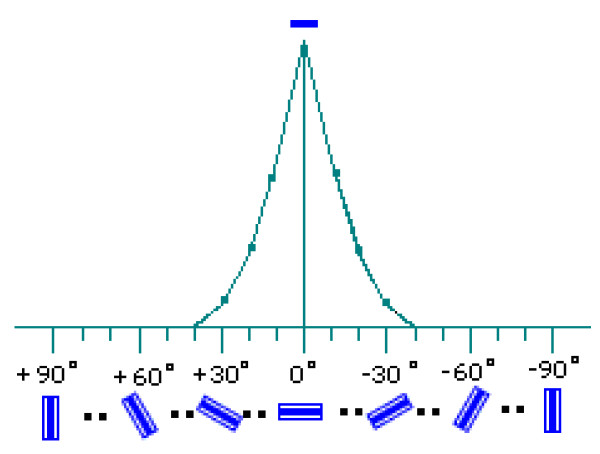
**Selective matching between *r*_*i*,*j *_(*a*) (a horizontal edge) and different receptive fields in functional columns**. The receptive field *g*_*i*,*j *_(*b*) with horizontal orientation response strongly.

Above process is illustrated in Figure 2, where the local patch is a horizontal edge and an optimal matching is found in receptive fields of horizontal orientation with a strong response. No optimal matches are found in receptive fields of other orientations, so we have weak responses.

Figure 2. Selective matching between *r*_*i*,*j*_(*a*) (a horizontal edge) and different receptive fields in functional columns. The receptive field *g*_*i*,*j*_(*b*) with horizontal orientation responses strongly.

It is worthy noting that the multi-scale processing function of a visual pathway is to guarantee a clear image at a proper resolution in V1. So, the optimal matching is related with some resolution. When the scale or resolution changes, the optimal matching may concentrate on different extents of details, or may include or exclude some details, as is determined by the circumstances when the vision system is "observing" the world.

### The whole matching between the retinal image and receptive field patterns in V1

In the previous section, we have described the local matching or detection (formula 3). Next we will analyze the image matching as a whole, to obtain a mathematical representation of the neural firing pattern in V1. It is understood that the topographical mapping from retinal image *R*(*x, y*) to V1 is actually a stimulating process of *R*(*x, y*) with respect to simple selective cells with features of orientation, bandwidth and so on. Receptive fields *G*(*x*, *y*) of activated neurons are combined to form the global firing pattern *Φ*(*x*, *y*) in the cortex, as the responding process of simple cell groups to the visual image. So, the firing process can be regarded as a global matching between *G*(*x*, *y*) and *R*(*x, y*) at the system level, and eventually responding pattern *Φ*(*x*, *y*) will be formed. Many methods can be used to measure the extent of matching. However, in order to ensure a minimal reconstruction error, we adopt the following measure:(5)

where *B *is the imaging region in V1.(6)

Let . In the optimal matching in region *B*, we will have *R*(*x*, *y*) = *G*(*x*, *y*), as is the case when the equal operation is adopted in formula (5) and *λ*_*RG *_reaches its maximum value *λ*_*MAX*_. Therefore, we can define the normalized matching coefficient *ρ*_*rg *_as follows:(7)

Obviously when *ρ*_*rg *_= 1, *G*(*x*, *y*) and *R*(*x*, *y*) reaches a complete match. In other words, the receptive fields of all activated neurons in V1 are combined to form the same responding pattern *Φ*(*x*, *y*) as the whole visual image. It shown that this process can be mathematically described as follows by multiplication of *R*(*x*, *y*) and *G*(*x*, *y*)(8)

It can be seen later, *R*(*x*, *y*) and *G*(*x*, *y*) can be expressed as matrix form, for this reason, the formula (8) is essentially an inner product operation, it is not only more elegant on the mathematical form, but also more clear on the neurobiological significance.

Additionally, we can see the normalized matching coefficient *ρ*_*rg *_is equivalent to  in the previous section.

### Determination of integral kernel function

In formula (8), the role played by receptive fields *G*(*x*, *y*) of cortical neurons is the same as the integral kernel in wavelet transform [46,47]. It can be described as oriented and bandwidth two-dimensional Gabor function *G*(*x*, *y*)_*λ,σ,θ,φ,γ *_[34-39](9)

where *γ *is the ratio of the length in the major axis direction to that of in minor axis direction, usually set to a constant 0.5; *σ *is derivative of Gauss, determining the size of receptive fields; *φ* is the phase, when *φ *= 0; *π*; *G*(*x*, *y*)_*λ,σ,θ,φ,γ *_is symmetric about the origin; when *φ *= -(*π*/2); (*π*/2), *G*(*x*, *y*)_*λ,σ,θ,φ,γ *_is anti-symmetric about the origin; Θ is the optimal orientation, and *λ *is the wavelength. These arguments should be determined by experimental results from morphology and biophysics, but the exact data are not available so far [48]. One plausible way is to set the arguments according to input image features in an input-driven topological mapping [2]. This will be explained in the last section.

Substituting (9) into (8) and considering the cortical responses to orientation and bandwidth properties, we replace *Φ*(*x*, *y*) with *Φ*(*x*, *y*)_,*λ,σ,θ,φ,γ*_(10)

It is also the inner product, because the formula can be also expressed as matrix form. The formulae (8) and (10), as the inner product, which shows such an important neurobiological fact, that is, in the visual pathway topographical mapping indicates accurate positioning of retinal image in the visual cortex, therefore, these primitives can only respectively activate cells which are in the corresponding locations in visual cortex. Since it is a one to one excitation, scanning and convolution is no longer needed.

### Comparison of inner product with convolution

For comparison, the convolution operation may be expressed as follows(11)

We know that convolution and cross-correlation operations are essentially filtering operations in the frequency domain, which is not needed for V1, because such a filtering operation would lead to loss of high-and low-frequency information from the retinal picture. The second reason is that the scan process in such operations (convolution and cross-correlation) is a calculation with a high cost (see the section of discussion in this paper, for detail), in which, *G*(*x*, *y*)_*λ,σ,θ,φ,γ *_should be taken as the template to scan the whole image *R*(*x*, *y*) from top to bottom and from left to right. Obviously, it is not an effective method, because this scanning will cause too many responses of corresponding cells and the energy cost is too great.

Mathematically, the discrete convolution of formula (11) can be expressed as(12)

**Figure 3:**
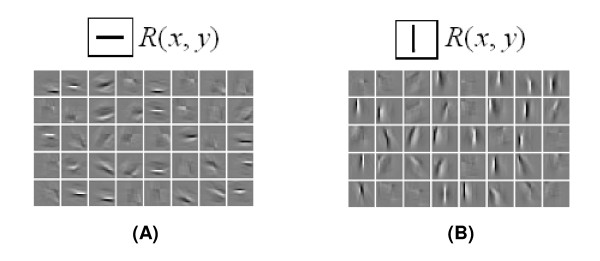
**Convolution operation for horizontal lines (A) and vertical lines (B) in image patches in R(*x, y*)**.

For some point (*x*_0_, *y*_0_) in an image, *G*(*x*_0 _- *k*, *y*_0 _- *l*)_*λ,σ,θ,φ,γ *_is moved around on the image (by changing *k *and *l*) to realize an optimal match between *Φ*(*x*_*k*_, *y*_*k*_) and *R*(*x*, *y*). The matched simple cell then is activated. Figure 3 shows an example, in which the visual image *R*(*x*, *y*) is a small horizontal or vertical line. Obviously, a horizontal line in *R*(*x*, *y*) excites numerous cells whose receptive fields have an orientation similar to a horizontal line, and a similar effect occurs for a vertical line. Such a calculation comes with a high costs in time and complexity.

Figure 3. Convolution operation for horizontal lines (A) and vertical lines (B) in image patches in *R*(*x*, *y*).

The use of the inner product reveals that neuron firing caused by a visual stimulus is in fact a simple function. This is a simpler neural computation than the cross-correlation and convolution operations, because it needs only multiplication between corresponding pixels of *Φ*(*x*, *y*)_*λ,σ,θ,φ,γ *_and *R*(*x*_*k*_, *y*_*k*_). It is worth pointing out that the product *R*(*x*_*k*_, *y*_*k*_)*Φ*(*x*_*k*_, *y*_*l*_)_*λ,σ,θ,φ,γ *_means that the retinal image *R*(*x*_*k*_, *y*_*k*_) excites all cortical cells and forms a global activity pattern *Φ *(*x*, *y*)_*λ,σ,θ,φ,γ *_in V1. Different visual stimuli will excite and form different activity patterns corresponding to that stimulus; the differences in activity patterns occur only in a topographically connected weight coefficient of the pixels of *R*(*x*_*k*_, *y*_*k*_) with the corresponding pixels of *Φ*(*x*, *y*)_*λ,σ,θ,φ,γ *_in a fully mapping neural computation. Generally, the weight coefficients corresponding to detailed image information are much smaller than those corresponding to contour and edge information. The intensity of the spike firing of simple cells excited by the details of the stimulus is also weaker than the intensity corresponding to contours and edges.

The inner product  in equation (8) reveals the collective calculation of a simple neuronal "on" or "off" function. From this, we can see that the calculation of the inner product is very well suitable to the visual system in that it satisfies the prerequisites of efficiency, simplicity, and robustness and also provides an optimal means of detection under the condition of least-mean-square-error reconstruction.

In fact, formula (9) reflects a specific wavelet transform on retinal image *R*(*x*, *y*) by basis function *G*(*x*, *y*)_*λ,σ,θ,φ,γ*_. This formula reflects the neural firing stimulated by the retinal image at the system level. Next we will discuss how to process visual images according to this formula. Two important problems will be discussed, that is, how to divide visual image *R*(*x*, *y*) according to structures and functions of the visual pathway and how to express the orientation selectivity of functional columns in V1 by two-dimensional wavelet function *G*(*x*, *y*)_*λ,σ,θ,φ,γ*_.

### Kronecker product in V1

Usually, visual image *R*(*x*, *y*) is independently transferred to LGN through 1 million ganglion cells, and then reaches the layers of 4*Cα *and 4*Cβ *in V1, and finally an image is reproduced in V1. Obviously, every image patch *r*_*i*,*j*_(*a*) is transferred through one channel. Suppose the number of channels is M × N, which means visual image *R*(*x*, *y*) is divided into *M *× *N *units. As pointed out in previous section, each patch is assumed to have the same size as the receptive field of a ganglion cell, namely, *a *= Δ*x *× Δ*y*. The area of the whole image is *A*. In every channel only one patch of [*R*_*i*,*j*_(*a*)]_*M *× *N*_, with local features, is transferred. All *r*_*i*,*j*_(*a*) are added to form [*R*_*i*,*j*_(*a*)]_*M *× *N*_.(13)

The receptive field of a neuron distributed in V1 is *g*_*i*, *j*_(*b*). After being stimulated by [*R*_*i*,*j*_(*a*)]_*M *× *N*_, a response pattern [*Φ*_*i*,*j*_(*b*)]_*M *× *N *_(a *M *× *N *matrix) is formed from theses receptive fields as:(14)

According to neurophysiology and neuroanatomy [43], cortical modules are densely distributed in V1, with approximately 10^3 ^modules; the area of each module is approximately 1.8 mm × 1.8 mm, containing two function columns for both left and right eyes. Thus, the area related with every function column *B*_*k*,*l *_(*s*) is 0.9 mm × 0.9 mm. At the system level, before adequate neurophysiological and neuroanatomical knowledge may be available, these function columns are assumed to have the same function and be composed of many receptive fields with different orientations and frequencies [3].

**Figure 4:**
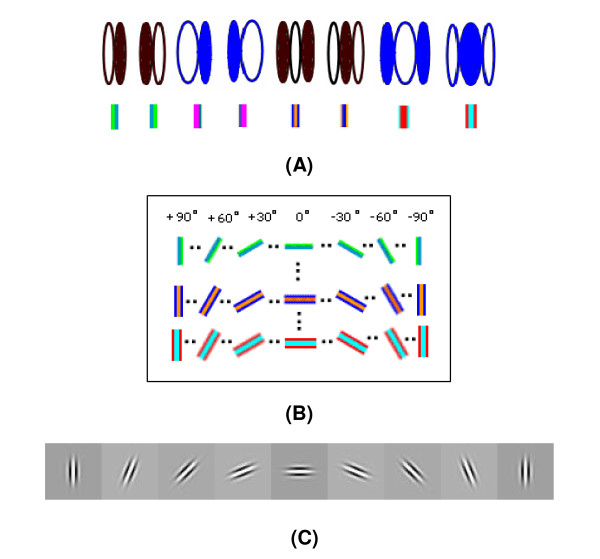
**Functional columns as basic information processing units**. (A) Eight representative types of receptive fields in function columns in V1; (B) Orientations range from 0° to 180° with a same interval of 10°; (C) An example of receptive field calculated by formula (9).

In this paper, the receptive fields of the function column can be represented as a matrix. As in Figure 4, each row of the matrix represents eighteen oriented receptive fields of the same type uniformly distributed from 0° to 180°. Each column of the matrix represents eight types of receptive fields (orthogonal Gabor of different frequencies) with a same orientation. So [*B*_*k*,*l*_(*s*)]_*K *× *L *_is made up of 144 elements *g*_*i*,*j *_(*b*) (*k *= 1,2,⋯, *K*; *l *= 1,2,⋯, *L*)(15)

Figure 4. Functional columns as basic information processing units. (A) Eight representative types of receptive fields in function columns in V1; (B) Orientations range from 0° to 180° with a same interval of 10°; (C) An example of receptive field calculated by formula (9).

**Figure 5:**
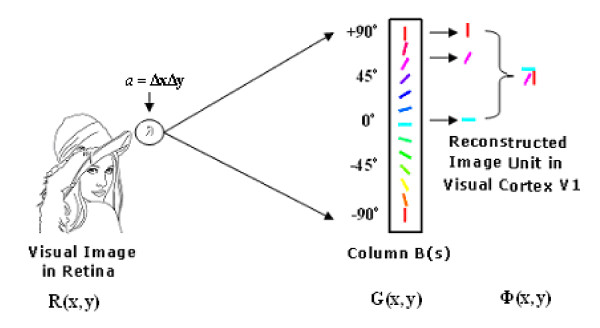
**Optimal matching between a patch (upper right corner of the hat) and receptive fields of specific orientations in cortical modules [*B*_*k*,*l *_(*s*)]_*K *× *L*_**.

Therefore, some edge or contour located at (*i*, *j*) in retinal image [*R*_*i*,*j*_(*a*)]_*M *× *N *_with area *a *will find a best match with the receptive field *g*_*i*,*j*_(*b*) of the same orientation and shape. When *ρ*_*rg  *_= 1, it means the patch at (*i*, *j*) in [*R*_*i*,*j*_(*a*)]_*M *× *N *_completely matches the cortical module [*B*_*k*,*l*_(*s*)]_*K *× *L *_with the specific orientation. Then the neuron is activated with the strongest response. The process of this image reconstruction is shown in figure 5.

Figure 5. Optimal matching between a patch (upper right corner of the hat) and receptive fields of specific orientations in cortical modules [*B*_*k*,*l*_(*s*)]_*K *× *L*_.

When all the *M *× *N *patches in retinal image [*R*_*i*,*j*_(*a*)]_*M *× *N *_simultaneously (in a parallel manner) activate topographically corresponding neurons, response pattern [*Φ*_*i*,*j*_(*b*)]_*M *× *N *_is formed in V1. At the system level, this process can be described by Kronecker Product.

Optimal responding of a neuron in V1 is actually reached through detection of cortical module [*B*_*k*,*l*_(*s*)]_*K *× *L *_with respect to a patch *r*_*i*,*j*_(*a*) in retinal image [*R*_*i*,*j*_(*a*)]_*M *× *N*_, which can be represented as a product of them, i.e. *r*_*i*,*j*_(2^*h *^*a*)[*B*_*k*,*l*_(*s*)]_*K *× *L*_(16)

Where |max for {*r*_*i*,*j*_(2^*h*^*a*)*B*_*k*,*l*_(*s*)| *k *= 1,2,⋯8; *l *= 1,2,⋯,18} means taking the maximum in [*r*_*i*,*j*_(2^*h*^*a*)*g*_1,1_(*b*)⋯,*r*_*i*,*j*_(2^*h*^*a*)*g*_1,18_(*b*);⋯,*r*_*i*,*j*_(2^*h*^*a*)*g*_8,18_(*b*)]. When *i *= 1,2,⋯, *M*; *j *= 1,2,⋯, *N*, it is equal to Kronecker product between [*R*_*i*,*j*_(*a*)]_*M *× *N *_and [*B*_*k*,*l*_(*s*)]_*K *× *L*_. The two matrixes are not necessarily of the same dimension. It can be represented as(17)

Where ⊗ denotes Kronecker product, |max for ∀{*r*_*i*,*j*_(2^*h *^*a*)*B*_*k*,*l*_(*s*)},  = 1 or *ρ*_*rϕ *_= 1 denotes the maximal of all products between *r*_*i*,*j *_(2^*h *^*a*) and *B*_1,1_(*s*), *B*_1,2_(*s*),⋯, *B*_8,18 _(*s*) (As the result of an improvement of signal-to-noise ratio, the noise is reduced). In a neurobiological sense, only stimuli with optimal orientation and frequency may activate the strongest response of simple cells in V1. Formula (16) represents the activated pattern corresponding to a typical image. This pattern can be represented as a matrix(18)

In formula (17), [*Φ*_*i*,*j*_(*b*)]_*M *× *N *_is the representation of retinal image [*R*_*i*,*j*_(2^*h *^*a*)]_*M *× *N *_in V1, which involves an essential difference with the traditional coding.

As is known, an image *I*(*x*, *y*) can be represented as a linear combination of orthogonal basis functions *ψ*_*i*,*j*_(*x*, *y*)(19)

where *a*_*i*,*j *_are weights. Obviously, the intersection of *a*_*i*,*j*_*ψ*_*i*,*j*_(*x*, *y*) is not null, i.e.(20)

**Figure 6:**
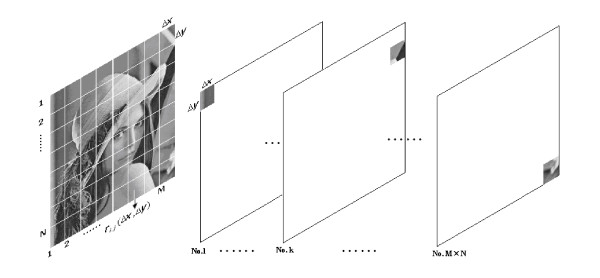
**Orthogonal division of a visual image**.

That is to say, the intensity at any location (*x*, *y*) in image *I*(*x*, *y*) is contributed by all basis functions *ψ*_*i*,*j*_(*x*, *y*) (*i *= 1,2,⋯, *M*; *j *= 1,2,⋯, *N*), so the computation can be highly complicated. In our case, the orthogonal division of input images makes every patch *r*_*i*,*j*_(*a*) orthogonal as shown in Figure 6; at the same time, the following condition is satisfied:(21)

Figure 6. Orthogonal division of a visual image.

Therefore, every patch *r*_*i*,*j*_(*a*) can be processed independently by functional columns. This is consistent with the neural mechanism of cortical information processing, and reduces computational complexity as well.

## Discussion

Currently, it is widely believed that simple cells densely distributed in V1 function similarly as a tiled set of selective spatio-temporal filters, while V1 carries out operations similar to the local complex Fourier transform. Theoretically, various kinds of neural processing about frequency, orientation, motion and other spatio-temporal operations can thus be performed [49,50].

In this way, the responding property *Φ*(*x*, *y*)_*λ,σ,θ,φ,γ *_in V1 is realized by a convolution between image *R*(*x*, *y*) and receptive fields *G*(*x*, *y*)_*λ,σ,θ,φ,γ *_[39].(22)

That is to say, *G*(*x*, *y*)_*λ,σ,θφ,γ *_is taken as a template to scan the whole image *R*(*x*, *y*) from above to bottom and from left to right. For example, if *G*(*x*, *y*)_*λ,σ,θ,φ,γ *_is a horizontal orientation receptive field, it will match to many edges with a similar orientation in *R*(*x, y*), so many cells in V1 are activated. The activated pattern *Φ*(*x*, *y*)_*λ,σ,θ,φ,γ *_is shown in Figure 3. A similar activated pattern corresponding to a vertical edge is shown in Figure 3(b). This is not an effective method for it stimulates too many responses of relative cells and costs a large amount of energy [51].

While in our case, in order to reconstruct retinal image [*R*_*i*,*j*_(*a*)]_*M *× *N*_, we only calculate activated pattern *ϕ*_*i*,*j*_(*b*) = *r*_*i*,*j*_(2^*h *^*a*)*B*_*k*,*l*_(*s*) of the receptive field stimulated by every patch *r*_*i*,*j*_(*a*) according to formula (15), and then the location of every patch is determined according to the topological mapping to V1 according to formulas (17) and (18). Finally, the whole activated pattern [*Φ*_*i*,*j*_(*b*)]_*M *× *N *_stimulated by image [*R*_*i*,*j *_(*a*)]_*M *× *N *_is obtained. Obviously, the related computation is much less complicated, which thus is more consistent with the multi-channel parallel processing mechanism in biology vision.

In order to compare computational complexity of the two ways of computation, we discretize formula (22) as(23)

Every element *ϕ*(*i*, *j*) in array [*Φ*_*i*,*j*_(*b*)]_*M *× *N *_involves *M *× *N *times of calculations, making the total calculations for all elements as *M*^2 ^× *N*^2^.

While in our case, the main computation is *ϕ*_*i*,*j*_(*b*) = *r*_*i*,*j*_(2^*h *^*a*)*B*_*k*,*l*_(*s*), so the total number of calculations are *M *× *N *× *K *× *L *(*K *<<*M*, *L *<<*N*). So the computation of Kronecker product is much less complicated than that of convolution.

We already noticed that a number of other researchers have developed linear-nonlinear models based on response properties of visual neurons [52,53], or on optimal nonlinear transformation [54]. In essence, they are a combination of linear filtering and divisive inhibition model; all of the models have been used to model the nonlinear responses of visual neurons and primary visual cortex. In terms of our proposed model, as already pointed out that theoretical analysis and simulated results show that at the system level, the inner product operator reflects the nature of the excitation of neurons in the cortex V1 by local characteristics of the external stimuli. This is also a plausible assumption for neural computation in the cortex V1. Therefore, it may have some reference value in investigations of neural mechanisms in visual information processing.

## Conclusions

It is understood that the retinal image must be in one-to-one correspondence with cortex V1, for all subsequent processing will extract information from V1 and the information kept in V1 is vital. Only in this way, the brain can perceive a vision through the retinal image with high fidelity. Neurophysiology shows that when a retinal image topographically projects to the visual cortex, corresponding neurons will be activated. The whole activated pattern is a copy of the retinal image with high fidelity. In view of signal processing, product *r*_*i*,*j*_(2^*h *^*a*)*B*_*k*,*l*_(*s*) means that receptive fields *g*_*i*,*j *_(*b*) in V1 are activated when stimulated by retinal image *r*_*i*,*j*_(*a*). Therefore, this operator is consistent with this neurobiology mechanism. It involves both the simple function of a single neuron and the population function of neuronal groups. *ϕ*_*i*,*j *_(*b*) is the local activated pattern corresponding to patch *r*_*i*,*j*_(*a*). Different stimuli produce different activated patterns of neuronal groups. The signals activated by details in visual stimuli are much weaker than those activated by contours. According to our understanding of the precise reconstruction of retinal images in the visual process, and based on multi-channel parallel processing features of the visual pathway, a visual image is divided into basic image units (patches) or primitives, which are topographically mapped onto the visual cortex by a one-to-one correspondence, by means of multiplication computing, features contained in image's primitives can be extracted by thousands of visual cortical modules in parallel and synchronously, where only an inner product (or Kronecker product)is needed, then an image will be formed in the primary visual cortex. This algorithm is simple, efficient and in line with the current knowledge about the neural mechanism of visual information processing, the mathematical description is also appropriate to the visual neural computation.

Visual information processing that is actually carried out in V1 is very important, but so far our knowledge of it at the system level remains inadequate [55-57] apart from Hubel and Wiesel's discovery [43] in the 1960s and Field and Olshausen's sparse coding theory [58] in the 1990s. Therefore, the neural computation model based on available knowledge about structure and function of V1 [59-65], presented in this paper may throw some light towards that direction, of course, will require further proof in neurobiology.

The next step of the studies on the visual information processing will be focussed on functional modules in cortex V1.

## Methods

**Figure 7:**
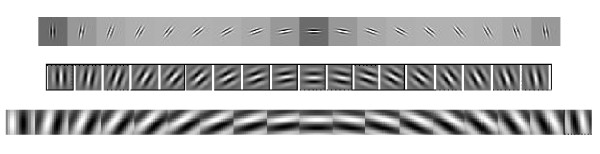
**Three representations of eight types of receptive fields in function columns in V1 calculated by Gabor function, in which orientations 0°, 10°, 20°, ⋯, 180° in turn**.

In numerical simulations, according to a resolution of 10°/50 μm, we divide orientations of receptive fields in function columns into 18 parts ranged from 0° to 180° with a same interval of 10°, i.e. 10°,200°,⋯,180°. Eight types of receptive fields are chosen in Gabor function *G*(*x*, *y*)_*λ,σ,θ,φ,γ *_according to formula (8); the result is shown in Figure 7.

Figure 7. Three representations of eight types of receptive fields in function columns in V1 calculated by Gabor function, in which orientations 0°, 10°, 20°, ⋯, 180° in turn.

**Figure 8:**
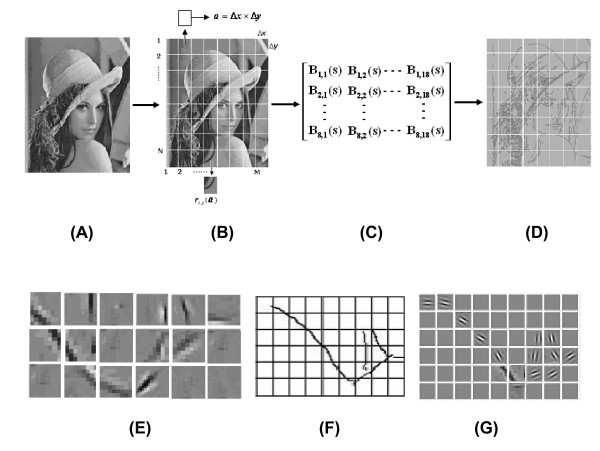
**Image reconstruction by topological mapping and Kronecker product**. (A) Source image Lenna; (B) Retinal image [*R*_*i*,*j*_(2^*h *^*a*)]_*M *× *N*_; (C) Receptive fields array *B*_*k*,*l*_(*s*) of functional columns in V1; (D) The whole activated pattern of receptive field image [*Φ*_*i*,*j*_(*b*)]_*M *× *N *_; (E),(F) and (G) A part of activated pattern [*Φ*_*i*,*j *_(*b*)]_*M *× *N *_in V1 calculated by formulas 14-16 (upper right corner of the hat).

And the arrays *B*_*k*,*l*_(*s*) are formed according to formula (13). Then the test image Lenna is divided into *M *× *N *patches according to formula (11). The activated pattern of every receptive field *ϕ*_*i*,*j *_(*b*) stimulated by a patch *r*_*i*,*j *_(*a*) is calculated by formula (14). The whole activated pattern [*Φ*_*i*,*j*_(*b*)]_*M *× *N *_stimulated by [*R*_*i*,*j*_(*a*)]_*M *× *N *_is processed by formulas (16) and (17). A simulated example of the whole activated pattern is shown in Figure 8.

Figure 8. Image reconstruction by topological mapping and Kronecker product (A)Source image Lenna; (B)Retinal image [*R*_*i*,*j*_(2^*h *^*a*)]_*M *× *N*_; (C) Receptive fields array *B*_*k*,*l*_(*s*) of functional columns in V1; (D) The whole activated pattern of receptive field image [*Φ*_*i*,*j*_(*b*)]_*M *× *N*_; (E), (F) and (G) A part of activated pattern [*Φ*_*i*,*j *_(*b*)]_*M *× *N *_in V1 calculated by formulas 14-16 (upper right corner of the hat).

**Figure 9:**
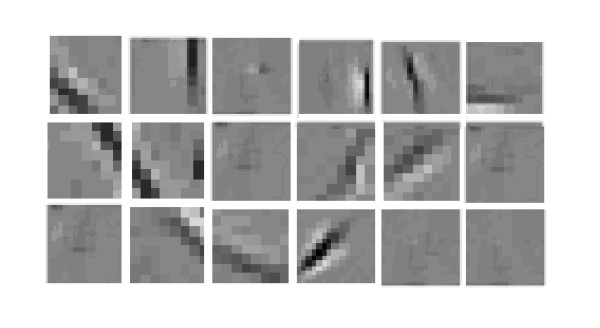
**Result (only of the upper right part of the hat shown) corresponding to the damaged function columns at *i *= 5, *j *= 9; *i *= 7, *j *= 2; *i *= 11, *j *= 15**.

In numerical experiments, we also consider the case when a part of cortex is damaged, i.e. functional columns at these locations do not function as they should in the image processing. We assume that the damaged positions are those with *i *= 5, *j *= 9; *i *= 7, *j *= 2; *i *= 11, *j *= 15. The corresponding result is shown in Figure 9. It can be seen that the damaged functional columns do not affect the integrity of the image, for other columns have made compensation for the damaged ones.

Figure 9. Result (only of the upper right part of the hat shown) corresponding to the damaged function columns at *i *= 5, *j *= 9; *i *= 7, *j *= 2; *i *= 11, *j *= 15.

## Authors' contributions

ZSN, ZQ took part in conceiving and designing the study, analyzed the data, and drafted the manuscript. JZ took part in designing the study and contributed to the data analysis. YGZ, YL took part in conceiving and designing the study. All authors have read and accepted the final version of the manuscript.
